# Co-morbid monogenic disorders at chromosome region 1q2: *LMNA*- and *FLG*-related disorders in a patient referred for assessment of joint hypermobility

**DOI:** 10.1007/s10577-025-09785-z

**Published:** 2025-10-30

**Authors:** Mayowa A. Osundiji, Adedamola O. Bello, Jennifer L. Hand

**Affiliations:** 1 Department of Clinical Genomics, Mayo Clinic, 200 First Street SW, Rochester, MN 55905 USA; 2 Pontiac General Hospital, Pontiac, MI 48341 USA; 3 Faculty of Medicine, Martinus University, Willemstad, Curacao, Netherlands; 4 Department of Dermatology, Mayo Clinic, 200 First Street SW, Rochester, MN 55905 USA; 5 Department of Pediatrics, Jacobs School of Medicine & Biomedical Sciences, University at Buffalo, Buffalo, NY 14203 USA

**Keywords:** Sequencing, Exome, Connective, Cardiovascular, Disorders, LMNA, FLG

## Abstract

The phenotypic similarities and genetic heterogeneity occurring in diverse forms of Ehlers Danlos Syndrome (EDS) subtypes and many heritable connective tissue disorders can pose a diagnostic challenge. In the wake of the growing applications of next‐generation sequencing technologies including exome and genome sequencing, opportunities for achieving definitive genetic diagnosis are increasingly arising. We present a 46-year-old man with joint laxity, recurrent joint subluxations, pelvic floor dysfunction, and postural orthostatic tachycardia syndrome (POTS), who was referred for EDS assessment. His medical history included morbid obesity requiring gastric bypass surgery, hearing loss, asthma, retinopathy, myopia, atrial septal defect, narcolepsy with cataplexy, polyneuropathy, folliculitis, lichen simplex chronicus, atopic dermatitis, and hypogonadism. His family history was significant for multiple first- and second-degree relatives who died from cardiac diseases including cases of childhood deaths. Physical examination showed joint laxity with Beighton score of 3/9, bilateral pes planus, hearing loss and macrocephaly. Exome sequencing revealed heterozygous variants *LMNA* c.1262 T > C p.L421P [classified as likely pathogenic], *FLG* c.2282_2285del p. S761Cfs*36 [classified as pathogenic], and *FLG* c.1501 C > T p. R501* [classified as pathogenic]. Mitochondria sequencing revealed a variant of uncertain significance (VUS), MT-ND2 m.5047 T > C p.V193A that is present at 9% heteroplasmy in blood. These findings show co-occurrence of pathogenic sequence variants in neighboring genes located in chromosome 1q2 region [*LMNA* and *FLG*] in a patient with features of hereditary connective tissue disorders. Our study highlights the capability of exome sequencing in achieving some actionable diagnosis in cases of co-morbid genetic disorders with overlapping and non-specific symptoms.

## Introduction

Genetic disorders of connective tissues encompass a heterogenous and growing list ofMendelian syndromes [e.g. Ehlers-Danlos syndrome (EDS), Marfan, Loeys-Dietz, osteogenesis imperfecta, spondyloepiphyseal dysplasia, Stickler syndrome, laminopathies, FLG-related disorders etc.] that can share some overlapping clinical features involving the musculoskeletal, cutaneous, cardiovascular, neurologic, and other systems. Of the hereditary connective tissue disorders, hypermobile EDS (hEDS) and hypermobility spectrum disorder (HSD) appear to be common (Alcorta-Sevillano et al. 2020; Baban et al. 2020). Referral for HSD and EDS subtypes assessment following findings of joint hypermobility, skin hyperextensibility, easy bruising and/or skin fragility occur frequently resulting in an increasing need for novel approaches to manage the clinical demand (Behera et al. 2025; Benarroch et al. 2021; Boriani et al. 2018). Discerning actionable hereditary connective tissue disorders from non-specific causes of joint hypermobility and skin diseases is crucial, although not always straightforward (Behera et al. 2025; Butler et al. 2022; Capell and Collins 2006; Carboni et al. 2013)(Chen et al. 2013).

Improvements in genetic testing technologies in the last few decades have resulted in an increased availability of exome and genome sequencing, which hold great potential to facilitate the diagnosis of monogenic disorders including hereditary connective tissue disorders (Colombi et al. 2015; de Toledo et al. 2020; Debinska 2021). LMNA-related disorders (laminopathies) (Debinska 2021; Decaudain et al. 2007; Desgrouas et al. 2020; Ferradini et al. 2021)and FLG-related diseases (Gersak et al. 2013; Gruber et al. 2011; He et al. 2019; Ho and Hegele 2019)are actionable hereditary connective tissue disorders (Holbrook and Byers 1989; Hoober and Eggink 2022; Huffmeier et al. 2008) that are associated with genes located in chromosome 1q2 region.

The LMNA gene encodes Lamin A and C (generated through alternative splicing), which are intermediate filament proteins that are present in the nuclear lamina (protein network lining the inner surface of the nuclear envelope) of cells and play pertinent roles in the cell’s structural stability, cellular motility, mechanosensing, cellular differentiation, gene regulation, DNA damage repair, telomere protection and chromosome organization (Irvine et al. 2011; Knight et al. 2022). Mutations in*LMNA*cause over 15 different forms of laminopathies, diseases mainly affecting mesenchymal tissues, manifesting with overlapping clinical phenotypes that can be suggestive of a continuum of related disorders (Decaudain et al. 2007; Kore et al. 2025; Lapp et al. 2014; Liu and Ikegami 2020). Laminopathies present with diverse phenotypes, including cardiomyopathies [e.g. LMNA-related dilated cardiomyopathy with conduction system disease, hand heart syndrome (Slovenian type) etc.], lipodystrophies [e.g. familial partial lipodystrophy type 2], muscular dystrophies (e.g. limb-girdle muscular dystrophy type 1B, Emery-Dreifuss muscular dystrophy, LMNA-related congenital muscular dystrophy), neuropathies (e.g. Charcot-Marie-Tooth disease type 2B), premature ageing syndromes (Hutchinson-Gilford progeria), bone diseases (e.g. Mandibulofacial dysplasia), skin disorders (restrictive dermopathy type 2), hypogonadism (e.g. Malouf syndrome) and metabolic syndrome (Desgrouas et al. 2020; Hoober and Eggink 2022; Huffmeier et al. 2008; Malashicheva and Perepelina 2021; Markova et al. 1993; Mosbah et al. 2020; Muller et al. 2009; Murphy-Ryan et al. 2010).

The FLG gene encodes the protein profilaggrin, a large polyprotein precursor (~ 400 kDa in humans) for the monomeric 37 kDa protein that is called filaggrin (Ogrodowczyk et al. 2014; Palmer et al. 2006). The name filaggrin was coined from filament-aggregating protein (Ogrodowczyk et al. 2014; Palmer et al. 2006; Pessente et al. 2022). Filaggrin plays important roles in the stratum corneum including the aggregation and collapse of keratin filaments, formation of the protective surface barrier, maintenance of skin hydration, pH regulation, microbial defense, and the overall integrity of skin tissue (Palmer et al. 2006; Quijano-Roy et al. 2008). Filaggrin deficiency is associated with ichthyosis vulgaris (MIM 146700), atopic dermatitis, and an increased risk of many other skin and allergic diseases (Gersak et al. 2013; Gruber et al. 2011; He et al. 2019; Ho and Hegele 2019; Reddy et al. 2017; Retterer et al. 2015, 2016). Here we describe the first documented case of co-morbid LMNA-related disorder and FLG-related disease, a discovery that was possible through exome sequencing.

## Materials and methods

The study was approved by the Institutional Research Ethics Board. We recruited Individual 1 for a research study involving the use of exome sequencing for diagnosis of adult patients with medical complexities that include connective tissue dysfunction, who are suspected of having undiagnosed genetic condition(s). Exome sequencing with mitochondria genome sequencing was performed using high-quality genomic DNA extracted from peripheral blood lymphocytes at GeneDx laboratory (Gaithersburg, MD) as previously described (Richards et al. 2015). Briefly, the DNA was enriched for the complete coding regions and splice site junctions across the human genome using a proprietary capture system developed by GeneDx. Paired-end sequencing of the enriched targets was performed on an Illumina platform. Bidirectional sequence reads were assembled and aligned to the reference sequences based on the NCBI RefSeq transcripts and the human genome reference sequence, GRCh37/hg19. Trimming of reads, alignment and mapping were performed via Illumina’s DRAGEN (Dynamic Read Analysis for GENomics) software (Ritelli and Colombi 2020). 98.7% of the exome covered at ≥ 10 sequence reads (10x). The mean depth of coverage of the reads was 92x.

Reported variants were confirmed, if necessary, by an appropriate orthogonal method [such as long-read sequencing, chromosomal microarray analysis, multiplex ligation-dependent probe amplification etc.]. Sequence variants were reported according to the Human Genome Variation Society (HGVS) guidelines. Variants were classified following the American College of Medical Genetics and Genomics (ACMG) and the Association for Molecular Pathology (AMP) guidelines for interpreting genetic variants identified in Mendelian disorders (Ritelli et al. 2018)using GeneDx laboratories’s custom-developed analysis tool (XomeAnalyzer) (Ritelli et al. 2020). The ACMG/AMP Mendelian gene variant stratification guidelines encompass 28 criteria that help clinical laboratories collate evidence level for benign or pathogenic impacts of variants identified in genes that are known to be associated Mendelian disorders. The evidence types and level of strength are classified with associated codes as stand‐alone for benign (BA), pathogenic very strong (PVS1), pathogenic strong (PS1-4), pathogenic moderate (PM1-6), or pathogenic supporting (PP1-5). The evidence types are summated to decide whether a Mendelian gene variant should be classified as PV, LPV, variant of uncertain significance (VUS), likely benign variant (LBV), or benign variant (BV) (Ritelli et al. 2018). Variant review via XomeAnalyzer encompassed nucleotide and amino acid annotations, analysis of variant type, interface with population frequencies databases [such as gnomAD (Rodriguez et al. 2009), in silico prediction tools, inheritance patterns, clinical scientific literature, functional and segregation studies etc. (Ritelli et al. 2020).

## Clinical report

**Fig. 1:**
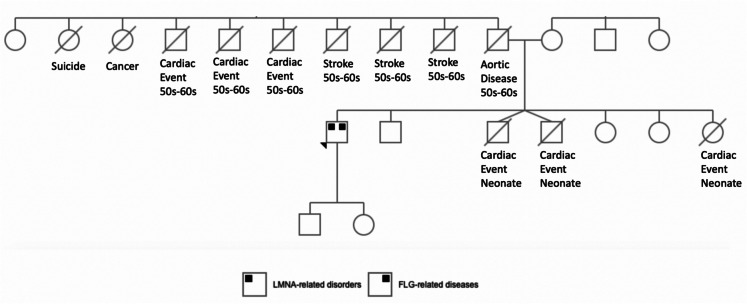
Pedigree of the patient’s family showing 4 first degree relatives who died in a context of cardiac and/or vascular events including 3 cases of childhood deaths. Also represented are 6 s degree relatives who died in their 50 s and 60 s from cardiovascular disease. The arrowhead points to the index patient

Individual 1 is a 46-year-old man, who was referred for evaluation of suspected EDS in a context of a longstanding history of joint laxity with recurrent joint subluxations and arthralgia. He reported a history of congenital talipes equinovarus with delayed gross motor milestones, severe atopic dermatitis, folliculitis, lichen simplex chronicus, asthma, and atrial septal defect that spontaneously resolved. The remainder of his perinatal, infant and childhood history were otherwise non-contributory. By adolescence, he developed obesity, bilateral sensorineural hearing loss, palpitations and episodes of blackouts requiring Holter monitoring and cardiology follow-up, myalgia, and muscle weakness. In early adulthood, he developed morbid obesity and type 2 diabetes mellitus. He underwent gastric bypass surgery, Roux En Y. He also had a history of knee surgery. He experienced other medical co-morbidities including narcolepsy with cataplexy, postural orthostatic tachycardia syndrome, pelvic floor dysfunction, axonal polyneuropathy (suggested by nerve conduction studies), electromyographic evidence of proximal myopathy and hypogonadism. With regards to his dermatologic history highlighted above, he reported a lifelong history of severe eczema. He explained that his armpit, groin, and other intertriginous areas felt sore very often and almost lifelong. His family’s medical history was remarkable for multiple family members who died from cardiac or vascular diseases. He reported a history of 4 first degree relatives who died in a context of cardiac and/or vascular events including 3 cases of childhood deaths. He had 6 paternal uncles who reportedly died from cardiac and/or vascular diseases (Fig. 1).

On physical examination, he had macrocephaly, joint laxity with Beighton score of 3/9, and bilateral pes planus. He elected to pursue singleton exome sequencing with mitochondria genome sequencing through GeneDx laboratory (Gaithersburg, MD). Exome showed heterozygous variants: *LMNA* c.1262 T > C p.L421P [classified as likely pathogenic], *FLG* c.2282_2285del p. S761Cfs*36 [classified as pathogenic], and *FLG* c.1501 C > T p. R501* [classified as pathogenic]. Mitochondria genome sequencing revealed a variant of uncertain significance (VUS), MT-ND2 m.5047 T > C p.V193A. This variant was found to be present at very low level heteroplasmy (9%) in blood.

## Discussion

We report the first documented case of co-morbid laminopathy and FLG-related disorders in a patient with joint laxity, cardiac disease and atopy. LMNA mutation carriers have increased risk of sudden death due to cardiac and vascular events including arrythmias, heart failure and thromboembolic disease (Ferradini et al. 2021; Sandilands et al. 2007, 2009). The proband had multiple first and second-degree family members who died from cardiac and/or vascular diseases of unclear etiology (Fig. 1). This case report underscores the importance of timely and accurate diagnosis of LMNA-related disorders to facilitate the clinical management of the associated cardiac risks. In the era of novel and upcoming therapies for FLG-related diseases (Schubart et al. 2021), our findings also highlight the significance of prompt detection of*FLG* mutations.

LMNA-related disorders and FLG-related disorders are rare genetic diseases associated with chromosome 1q2 region, however the sequence variants *LMNA* c.1262 T > C p.L421P, *FLG* c.2282_2285del (2282del4) p. S761Cfs*36, and *FLG*c.1501 C > T p. R501* (R501X), have been described in the clinical scientific literature (Desgrouas et al. 2020; Gruber et al. 2011; Silfeler et al. 2014; Smith et al. 2006).*LMNA*L421P was previously observed in a family with severe obesity (affecting 7 individuals, proband and 1st-degree relatives), type 2 diabetes mellitus, metabolic syndrome, hypertension, lower limb pain and weakness (Desgrouas et al. 2020; Tao et al. 2021). Although these published clinical manifestations of*LMNA* L421P overlap with our patient’s clinical presentation (Table 1), the complete range of phenotypes associated with *LMNA* L421P are yet to be fully defined. It remains possible that hypogonadism, axonal neuropathy, congenital talipes equinovarus, palpitations and episodes of blackouts in our patient may represent additional multisystemic manifestations of *LMNA*L421P. Hypogonadism occurs in LMNA-related Malouf syndrome (Thyssen et al. 2012). Congenital talipes equinovarus (Thyssen et al. 2013), muscle weakness (including pelvic muscles) (Desgrouas et al. 2020), neuropathy (Ferradini et al. 2021), cardiovascular malformation (Tinkle et al. 2017)(Ferradini et al. 2021), cardiac conduction diseases (Thyssen et al. 2013) are known LMNA-related phenotypes. The molecular mechanisms underpinning*LMNA* L421P laminopathy have not been completely elucidated. Studies by Yang and colleagues suggest that *LMNA*L421P may result in possible impairment of lamin A to Nuclear Envelope Spectrin Repeat Proteins-2 (Nesprin-2) interactions and cause Linker of Nucleoskeleton and Cytoskeleton (LINC) complex alterations in the nuclear envelope (Yang et al. 2013).

**Table 1:** A comparison of the features of our patient with recognized manifestations of LMNA and FLG-related disorders

Relevant Systems	Features of LMNA-Related Disorders in the Proband Harboring *LMNA* c.1262 T > C p.L421P	Features of FLG-Related Disorders in the Proband Harboring *FLG* c.2282_2285del p. S761Cfs*36, and c.1501 C > T p. R501*	Unexplained Features
Cardiovascular	Atrial septal defect, hypertension, palpitation, blackout, postural orthostatic tachycardia syndrome		
Nervous	Muscle weakness, axonal neuropathy, proximal myopathy		Sensorineural hearing loss
Skin and Allergy		Atopic dermatitis, lichen simplex chronicus, asthma	Psoriasis
Musculoskeletal	Congenital talipes equinovarus, joint laxity, pelvic floor dysfunction, muscle pain		Macrocephaly, narcolepsy with cataplexy, rheumatoid arthritis
Endocrine and metabolic	Obesity, type 2 diabetes mellitus, hypogonadism		

The 2282del4 and R501X founder mutations are prevalent in European and Asian populations and are the most common *FLG*mutations in the clinical scientific literature (Ho and Hegele 2019). Although it is unclear whether the FLG loss of function mutations identified in this case report [2282del4 and R501X] are in*cis* or *trans*, heterozygotes are known to manifest the phenotypes (Gruber et al. 2011). FLG-related disorder explains the patient’s history of severe atopic dermatitis, lichen simplex chronicus, and asthma. Nevertheless, the*FLG* and *LMNA* variants do not fully explain the patient’s clinical presentation (Table 1). The *FLG*variants do not readily explain his history of hearing loss (Smith et al. 2006), psoriasis (Yang et al. 2022)or rheumatoid arthritis (Yew et al. 2021). There are emerging roles for FLG and LMNA in other disease processes (Silfeler et al. 2014). Although a variant was detected in the mitochondria genome, MT-ND2 m.5047 T > C p.V193A, the variant was found to be present at very low level heteroplasmy (9%) in blood and classified as VUS.

An area of strategic interest with this patient (and potentially with his family members) was the difficulty in reaching a genetic diagnosis. The diagnostic odyssey lasted several decades. The initial suspicions of possible genetic underpinnings reportedly began in early childhood in the context of his history of clubfoot and delayed motor milestones. With the findings of joint laxity in adulthood, possibility of hereditary connective tissue disorder(s) was raised by his healthcare providers. As his phenotype evolved in adulthood with diverse co-morbidities (including morbid obesity) in the setting of unrecognized LMNA-related disorders and FLG-related diseases, the potential for genetic diagnosis became less appreciated with suspicions of more common environmental etiologies. The family history of multiple fatal cardiovascular events and the patients ongoing history of joint laxity triggered a referral to our genetics clinic. Although referrals for HSD and hEDS are common, the patient’s presentation highlights the importance of clinical risk assessment for potential diagnostic benefits of genetic testing. The diagnosis of LMNA-related disorders and FLG-related diseases prompted further cardiac, dermatologic, neurologic, and endocrine management plans for the patient thus affirming the clinical utility of exome (You et al. 2017).

## Data Availability

The authors confirm that the data supporting the findings of this study are available within the article.

## Abbreviations

EDS: Ehlers-Danlos syndrome
hEDS: Hypermobile Ehlers-Danlos syndrome
HSD: Hypermobility spectrum disorder
VUS: Variant of unknown significance
Nesprin: Nuclear Envelope Spectrin Repeat Proteins
LINC: Linker of Nucleoskeleton and Cytoskeleton
